# Sympathetic ophthalmia after 27-G pars plana vitrectomy

**DOI:** 10.1186/s12886-021-01961-z

**Published:** 2021-05-02

**Authors:** Yasuyuki Takai, Yoshihito Sakanishi, Masahiro Okamoto, Nobuyuki Ebihara

**Affiliations:** Department of Ophthalmology, Juntendo University Urayasu Hospital, 2-1-1 Tomioka, Urayasu City, Chiba, 279-0021 Japan

**Keywords:** Sympathetic ophthalmia, Vitrectomy, Uveitis

## Abstract

**Background:**

Sympathetic ophthalmia (SO) is a bilateral diffuse uveitis that can arise after ocular trauma or ocular surgery in the inciting eye. Pars plana vitrectomy (PPV) is one of the risk factors for SO. Several reports have described SO developing after 23- and 25-G PPV, but none have described SO occurring after 27-G PPV. We describe herein a case of SO after 27-G PPV for rhegmatogenous retinal detachment.

**Case presentation:**

A 42-year-old woman presented with visual disturbance in the right eye. Best-corrected visual acuity (BCVA) was 6/200 in the right eye. Fundus examination revealed off-macula retinal detachment with retinal tears at both ends of retinal lattice degeneration at the temporal-oven peripheral retina of the right eye. We therefore performed 27-G sutureless PPV on the right eye. After 12 days, the retina was reattached, and BCVA improved to 6/30 in the right eye. Fifteen days postoperatively, she experienced headache and reduced vision in both eyes. Symptoms gradually worsened, and she visited our hospital 21 days postoperatively. BCVA was 6/30 in the right eye and 6/15 in the left eye. Slit-lamp examination revealed uveitis in the anterior chambers of both eyes, and fundus examination showed papillitis and subretinal detachment at the posterior poles of both eyes. Optical coherence tomography revealed subretinal fluid in the maculae of both eyes and fluorescein angiography showed multiple hyperfluorescent leakage sites in the retinal pigment epithelium. Cerebrospinal fluid examination showed pleocytosis and human leukocyte antigen testing showed expression of the DR04 phenotype; therefore, the patient was diagnosed with SO. She was treated with steroid therapy, and her visual disturbance subsided and the subretinal fluid improved as well. Her BCVA was 6/15 for the right eye and 6/5 for the left eye 93 days after the initial surgery.

**Conclusion:**

The present case shows that even if the sclerotomy site of 27-G PPV is small, there is still a risk of SO occurring in the eyes of patients who underwent transconjunctival vitrectomy. Ophthalmologists should recognize SO as complication of 27-G PPV and carry out proper management as early as possible.

## Background

Sympathetic ophthalmia (SO) is a bilateral diffuse type of uveitis, which occurs after ocular trauma or ocular surgery in inciting eye [1]. The etiology of SO is still unknown, but it seems to occur as a result of autoimmune and inflammatory response to ocular antigens such as melanocytes, soluble fractions from the outer segments of photoreceptor cells [1]. SO may present with intraocular findings similar to those of Vogt-Koyanagi-Harada disease (VKH), and the most notable sign of SO is a granulomatous anterior chamber reaction or subretinal detachment in the posterior pole of the eye [1, 2].

Recent advances in microsurgical techniques have led to the increased performance of transconjunctival sutureless vitrectomy using micro instruments in the treatment of retinal diseases [3]. This has produced greater anatomical and functional success in cases of rhegmatogenous retinal detachment (RRD); faster wound-healing, decreased operation time, improved patient comfort, and less postoperative inflammation with early visual recovery have been reported. The recently introduced 27-G pars plana vitrectomy (PPV) has been gaining popularity, and use of this technique may lead to safer, less invasive surgeries [4].

The occurrence of SO following multiple intraocular surgeries has been reported, such as cataract extraction, iridectomy, and retinal surgeries [1]. PPV has been implicated in cases of SO, and several reports have described SO after 23- and 25-G PPV [5], but no previous reports have described SO after 27-G PPV. We describe herein a case of SO that occurred after 27-G PPV for RRD.

## Case presentation

A 42-year-old woman with no history of ocular diseases or conditions presented to our hospital complaining of visual disturbance in the right eye for the past 3 days. Best-corrected visual acuity (BCVA) was 6/200 for the right eye and 6/5 for the left eye. Slit-lamp examination showed no inflammation in the anterior chambers of both eyes. Fundus examination revealed off-macula retinal detachment, with retinal tears at both ends of the retinal lattice degeneration at the temporal-oven peripheral retina of the right eye. She underwent 27-G sutureless PPV with chandelier-assisted, endolaser photocoagulation, and sulfur hexafluoride gas injection. Postoperatively, she was treated with topical steroid (0.1% betamethasone). Postoperative inflammation was only mild in the anterior chamber and gradually decreased. Twelve days postoperatively, the retina was reattached. BCVA was 6/30 in the right eye, and the patient was discharged.

**Fig. 1:**
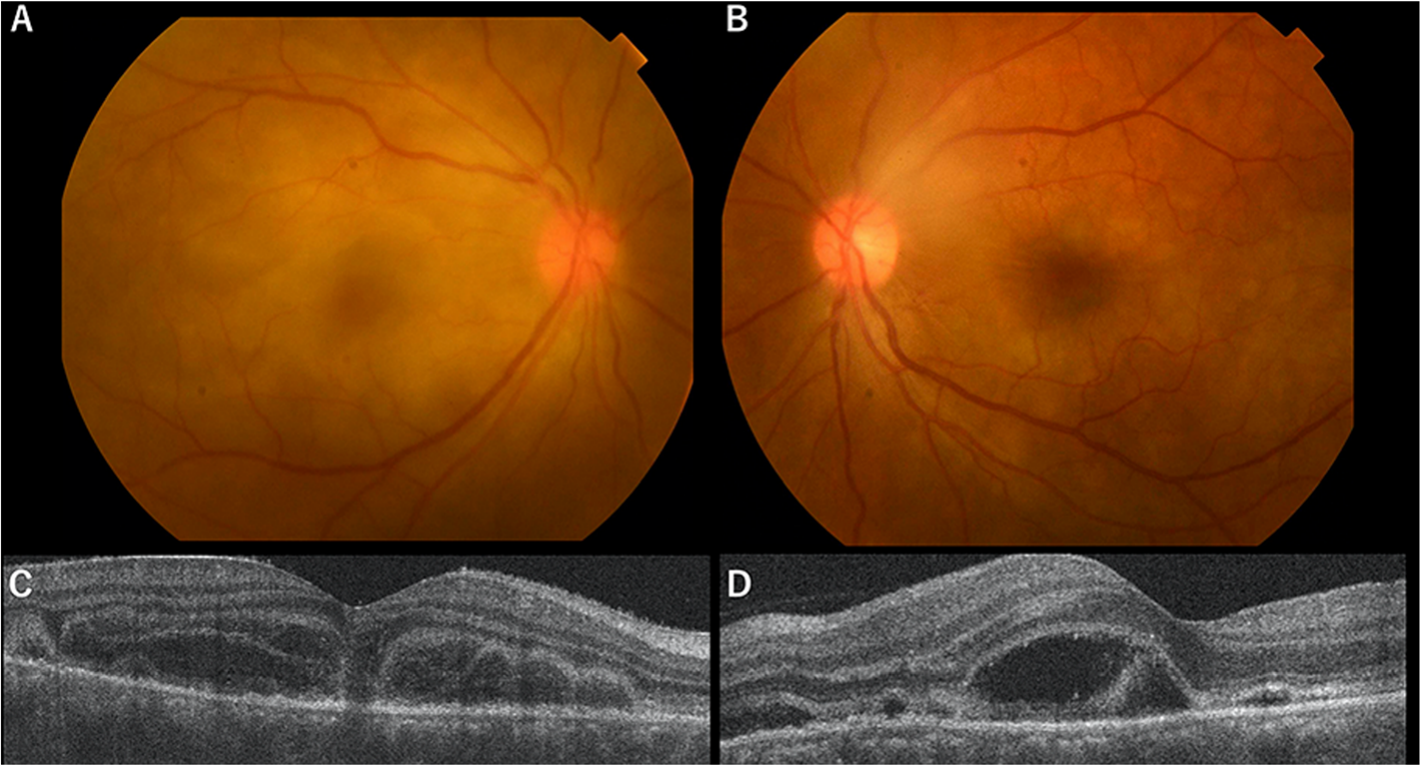
Fundus examination shows papillitis and subretinal detachment in the posterior poles of both eyes (**a**: right eye; **b**: left eye). Optical coherence tomography reveals subretinal fluid in the maculae of both eyes (**c**: right eye; **d**: left eye)

**Fig. 2:**
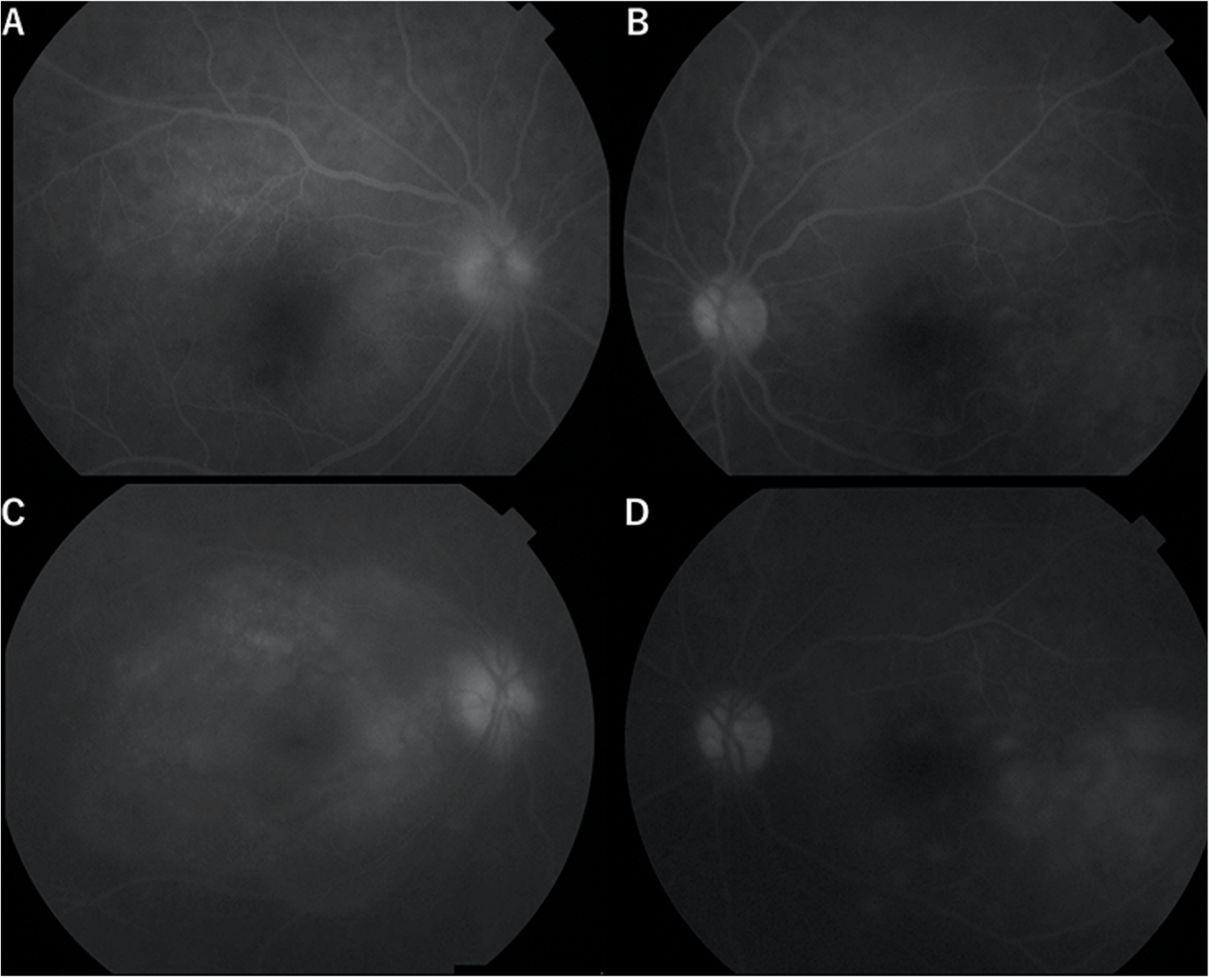
Fluorescein angiography shows multiple hyperfluorescent leakage sites in the retinal pigment epithelium during the early phase (**a**: right eye; **b**: left eye), and pooling of fluorescence in the area of exudative retinal detachment during the late phase (**c**: right eye; **d**: left eye)

Fifteen days postoperatively, she started experiencing headache and diminishing vision in both eyes. Symptoms gradually worsened, and she visited our hospital 21 days postoperatively. BCVA was 6/30 for the right eye and 6/15 for the left eye. Slit-lamp examination revealed ciliary injection, keratic precipitates, cells in the anterior chamber of both eyes, and posterior synechiae in the right eye. Fundus examination revealed papillitis and subretinal detachment at the posterior poles of both eyes (Fig. 1a, b). Optical coherence tomography (OCT) revealed subretinal fluid in the maculae of both eyes (Fig. 1c, d). Fluorescein angiography showed multiple sites of hyperfluorescent leakage in the retinal pigment epithelium during the early phase, and pooling of fluorescence into the area of exudative retinal detachment during the late phase (Fig. 2). Blood tests showed no elevated inflammatory response and no findings suggestive of auto-immune diseases such as sarcoidosis or vasculitis or infections such as tuberculosis, syphilis or fungi. Cerebrospinal fluid examination showed elevated mononuclear cells (136/μL). Human leukocyte antigen testing showed expression of the DR04 phenotype, so we diagnosed the patient with SO.

**Fig. 3:**
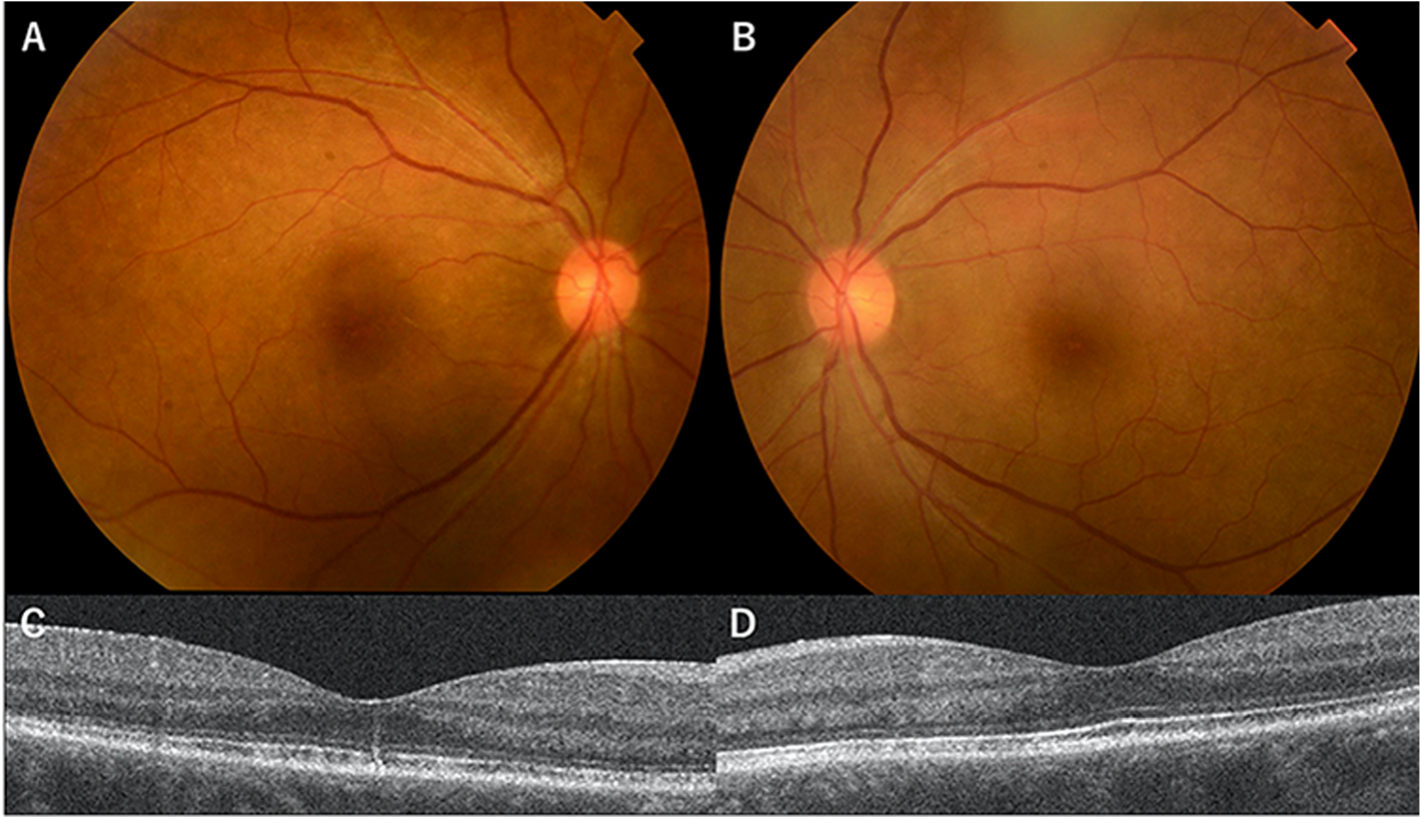
Fundus examination (**a**: right eye; **b**: left eye) and optical coherence tomography (**c**: right eye; **d**: left eye) show improvement of the subretinal fluid after steroid treatment

She was treated with steroid pulse therapy (methylprednisolone at 1000 mg/day for 3 days) and oral prednisolone (60 mg/day), in addition to intensive topical steroids (0.1% betamethasone). Visual disturbance subsided, and subretinal fluid improved (Fig. 3a–d). The dosage of oral prednisolone was gradually reduced. The patient was discharged 28 days after readmission to our hospital. She received repeated courses of low-dose oral steroid medication, and BCVA improved to 6/10 for the right eye and 6/5 for the left eye by 95 days after the initial surgery. Thereafter, steroids were tapered off, and the patient has remained recurrence-free for 1 year.

## Discussion

To our knowledge, no previous reports have described SO in 27-G PPV. In the present case, no findings suggested other uveitis such as viral infection, rheumatoid vasculitis or sarcoidosis. Findings from fluorescein angiography and OCT were indicative of uveitis, and she presented with meningitis, which has clinical features resembling those of VKH disease. As reported by Read et al., the only feature that reliably differentiates SO from VKH disease is a history of a penetrating wound [6], a criterion met by the history of PPV in this case. Although 27-G PPV permits the use of smaller incisions and is less invasive than previous PPV systems, a risk of SO occurring after the procedure remains.

Leakage of antigen from sclerotomy sites may lead to SO, because suturing at the site is not performed in 27-G PPV. Immunogenic risks of PPV are likely attributable to increased retinal manipulation and breakdown of antigens such as components of melanocytes and retinal antigens. The loss of scleral integrity allows intraocular autoantigens to access the conjunctival lymphatics, and antigens move to the regional lymph nodes, resulting in a cell-mediated immune response [7]. In fact, the risk of SO occurring after vitrectomy is nearly twice that of external retinal detachment repair [7]. Concerns that 23- and 25-G transconjunctival sutureless vitrectomies may be associated with an increased incidence of wound leak are also growing, which would mean that penetration of the sclera may raise the risk of SO. Although 27-G PPV is much less invasive, complications such as endophthalmitis can still occur [8]. Use of 25-G PPV may result in a higher rate of postoperative endophthalmitis than 20-G PPV [9]. Given the fact that the procedure involves sutureless vitrectomy and requires time for complete wound closure, SO may also occur.

**Fig. 4:**
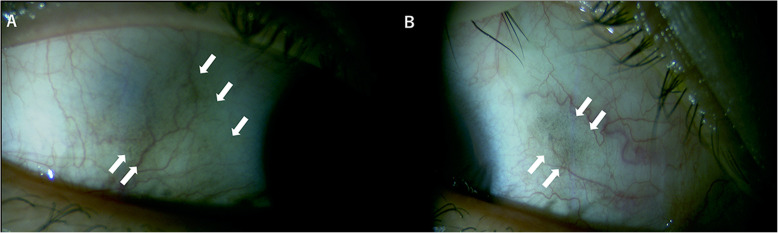
On postoperative examination, conjunctival pigmentation is apparent at the sclerotomy site in the right eye

In the present case, postoperative examination revealed conjunctival pigmentation at the sclerotomy site in the right eye (Fig. 4a, b). This may be related to subclinical uveal incarceration and presentation of antigens. Park et al. showed that patients with conjunctival pigmentation after 23-G PPV presented with SO signs similar to those in the present case [10]. Investigation using histological techniques identified the pigmented materials as macrophages that included melanin [10]. Rao et al. demonstrated induction of uveitis through injection of retinal antigens with adjuvants under the conjunctiva, but not into the eye, indicating that immune reactions may occur in conjunctival lymphocytes [11]. Sutureless vitrectomy, even 27-G PPV, can increase the chance of intraocular antigens entering the conjunctival lymphatics, which can therefore induce the occurrence of SO.

Postoperative steroid is effective to reduce postoperative inflammation [12], but proved unable to inhibit the development of SO in the present case. Bonfiglio et al. reported that postoperative oral prednisolone reduces the incidences of postoperative proliferative vitreoretinopathy and subretinal fluid [13], and so may suppress the postoperative inflammation or incidence of SO. Predicting the development of SO in patients with first-time RRD is difficult; thus, if a patient with a history of SO requires operation due to RRD, postoperative oral prednisolone use may be considered.

In cases of SO, prompt diagnosis and timely management are necessary to salvage vision [14]. SO occurs within 1 year after trauma or surgery in 90% of patients, and not only intraocular inflammation, but also systemic symptoms such as meningitis and hearing loss can occur [13]. Although 27-G PPV uses smaller incisions and is less invasive, SO may still arise. While SO is a rare disease, careful examination and prompt management are recommended.

## Conclusion

The present case shows that patients who have undergone 27-G PPV carry a risk of developing SO. The etiology of SO remains unclear, so careful observation of disease progression and starting treatment as early as possible are necessary to ensure good visual outcomes.

## Data Availability

Data for this case report were collected by chart reviews of the patient’s electronic medical record, which is not publicly available because of privacy considerations.

## Abbreviations

BCVA: best-corrected visual acuity
OCT: optical coherence tomography
PPV: pars plana vitrectomy
RRD: rhegmatogenous retinal detachment
SO: sympathetic ophthalmia
VKH: Vogt-Koyanagi-Harada
